# Optical Fresnel zone plate flat lenses made entirely of colored photoresist through an i-line stepper

**DOI:** 10.1038/s41377-024-01725-6

**Published:** 2025-01-16

**Authors:** Ryohei Yamada, Hiroyuki Kishida, Tomohiro Takami, Itti Rittaporn, Mizuho Matoba, Haruyuki Sakurai, Kuniaki Konishi

**Affiliations:** 1 Institute for Photon Science and Technology, The University of Tokyo, Tokyo, Japan; 2 JSR Corporation, Tokyo, Japan; 3 JSR-UTokyo Collaboration Hub, CURIE, Tokyo, Japan

**Keywords:** Metamaterials, Micro-optics

## Abstract

Light manipulation and control are essential in various contemporary technologies, and as these technologies evolve, the demand for miniaturized optical components increases. Planar-lens technologies, such as metasurfaces and diffractive optical elements, have gained attention in recent years for their potential to dramatically reduce the thickness of traditional refractive optical systems. However, their fabrication, particularly for visible wavelengths, involves complex and costly processes, such as high-resolution lithography and dry-etching, which has limited their availability. In this study, we present a simplified method for fabricating visible Fresnel zone plate (FZP) planar lenses, a type of diffractive optical element, using an i-line stepper and a special photoresist (color resist) that only necessitates coating, exposure, and development, eliminating the need for etching or other post-processing steps. We fabricated visible FZP lens patterns using conventional photolithography equipment on 8-inch silica glass wafers, and demonstrated focusing of 550 nm light to a diameter of 1.1 μm with a focusing efficiency of 7.2%. Numerical simulations showed excellent agreement with experimental results, confirming the high precision and designability of our method. Our lenses were also able to image objects with features down to 1.1 μm, showcasing their potential for practical applications in imaging. Our method is a cost-effective, simple, and scalable solution for mass production of planar lenses and other optical components operating in the visible region. It enables the development of advanced, miniaturized optical systems to meet modern technology demand, making it a valuable contribution to optical component manufacturing.

## Abstract (web summary)

The specially developed color resist enables low-cost, mass-production of visible light Fresnel zone plates using only exposure and development through an i-line stepper used in the semiconductor industry.

## Graphical abstract

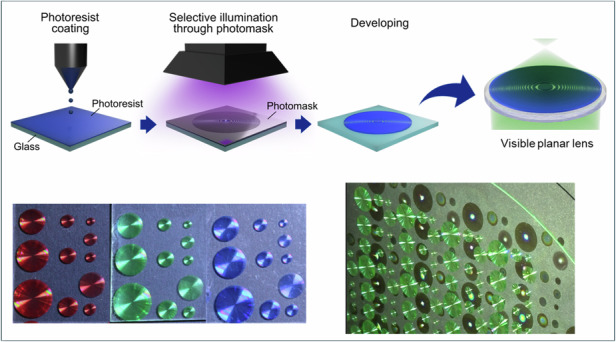

## Introduction

Light manipulation and control are a cornerstone in various contemporary optical technologies. As such technologies mature and become more miniaturized, the size constraints for their constituent optical components inevitably become more stringent. An indispensable basic component in optical systems is the lens. Traditional lenses utilize refraction to gather or focus light, but as they require a certain thickness to function, their size often becomes a limiting factor in the miniaturization of optical systems. Thus, there has been increasing demand for a flat lens to overcome these constraints, the most prominent technology of which is the metasurface^1–7^. Metasurfaces are optical components consisting of sub-wavelength structures; they utilize the microscopic optical response of tailored sub-wavelength structures to modulate the amplitude and/or phase of the transmitted light. By carefully engineering the spatial distribution of such tailored structures, optical functions, such as light focusing, can be realized. Many recent papers have demonstrated the fabrication of such new flat lenses using metasurfaces, which are then often called metalens^8–29^.

To fabricate metalenses that function in the visible region, structures on the order of a few hundred nanometers must be fabricated. Additionally, for many metalenses, dielectric structures with high aspect ratios must be fabricated to achieve a 2π phase difference; this requires high-resolution lithography and highly specialized dry-etching techniques. For instance, in the case of a visible metalens formed with TiO_2_ nanostructures, it is necessary to create pillars with diameters ranging from 100 nm to 220 nm and a height of 600 nm. It is required to form nanostructures with an aspect ratio of 6^11^. Many other metalenses have been reported, but they similarly require structures with a high aspect ratio^8–22^. Fabricating such high-aspect-ratio sub-wavelength structures typically uses electron beam lithography and dry etching technologies^16^. More recently, innovative techniques have been proposed to expand fabrication techniques, such as nanoimprint lithography^23,24^, two-photon polymerization printing^25^, and diffractive optical lenses produced by grayscale laser exposure^26^. Some of these approaches for realizing flat lenses do not require high aspect ratios^25,26^. Methods aimed at enhancing the resolution of photolithography, such as maskless optical projection nanolithography, have also been proposed^27^. As a result, flat lenses are being actively researched and developed using various methods to improve their performance.

While research studies are thus being carried out to improve the performance of metalenses, adapting these methods to mass production is still another challenge. The advanced microfabrication techniques described above, including electron beam lithography, are difficult to be used in industrial mass production to achieve large-area fabrication due to fabrication time and cost issues. In addition, millimeter- or centimeter-scale flat lenses are required for industrially important applications such as mobile phone lenses, cameras, and telescopes. In this regard, there is no doubt that the most competitive microfabrication method for mass production currently used in industry is photolithography using steppers. Steppers are widely used in the semiconductor industry to fabricate integrated circuit chips. Seminal works by Capasso and his colleagues successfully fabricated metalenses on the entire surface of a 4-inch wafer using deep ultraviolet (DUV) photolithography using a stepper, paving the way for the mass production of flat lenses by adopting conventional semiconductor fabrication processes^28,29^. However, even in this approach, advanced post-processing, such as high-aspect dry etching, is required after photolithography. Ultimately, it would be most desirable to be able to make flat lenses using only a stepper, paving a straightforward path towards mass-production compatibility.

The Fresnel zone plate (FZP) is a diffractive optical element consisting of a series of concentric rings with alternating transparent and opaque regions which acts as a diffraction grating, causing constructive interference at certain points to focus the incident waves^30,31^. Importantly, the FZP does not require high-aspect structures: while metalenses require structures with an aspect ratio of 8, the thickness of an FZP only depends on the amount of material required to block or absorb light in the opaque region, and therefore, can be less than 1. For this reason, the FZP structure is a strong candidate for mass-producible flat lenses with a stepper only.

In this study, we demonstrate the fabrication of visible FZP planar lenses using a very simple process with a special photoresist (color resist) which requires only coating, exposure, and development with an i-line stepper widely used in standard semiconductor microfabrication processes and without etching or other post-processing. The color resist is an ultraviolet-curable resin that contains an absorbent agent to shield specific wavelengths of visible light. The uncured parts that have not been exposed to ultraviolet light can be dissolved in an alkaline developer, thereby allowing the formation sub-microstructures with absorption characteristics. Because the color resist is highly absorbing at certain wavelengths, the microscopic color-resist pattern can be made to function as an amplitude-type diffractive optical element, including the FZP. In our experiments, we succeeded in mass-fabricating FZP lenses onto the surface of 8-inch silica glass wafers. The minimum line width of the fabricated structures was 1.1 μm. We evaluated the focusing properties of our lens and found that we could focus 550 nm light to a diameter of 1.1 μm with a focusing efficiency of 7.2% evaluated at an area of 3 times the FWHM. We compared results with simulations, finding excellent agreement, demonstrating that not only is our method very simple, but quantitatively designable. We lastly demonstrated imaging with the fabricated lens, successfully imaging objects with features down to 1.1 μm resolution. The developed method demonstrates one of the simplest practical methods for fabricating planar lenses and is therefore a powerful and cost-effective candidate for the mass-production of planar lenses operating in the visible region. It also possesses powerful potential as a general platform for the fabrication of other transmission-type optical components utilizing interference from a planar surface.

## Results

### Design and fabrication of FZP lenses

We first detail the design and fabrication of our FZP lens. The FZP lens is a type of planar diffractive lens with repeating concentric opaque and transparent regions^32^. The widths of the regions are engineered so that light diffracted from the transmissive region all constructively interfere at a designed focal point. For an FZP lens designed to focus light of wavelength ${\lambda }_{d}$ with a central opaque region, the $n$-th radii (${r}_{n}$) at which an opaque-to-transmissive (for odd $n$) or transmissive-to-opaque (for even $n$) boundary occurs can be given by the following equation^32^:

1 $${r}_{n}=\sqrt{n{\lambda }_{d}\left(f+\frac{n{\lambda }_{d}}{4}\right)}$$

where $f$ is the focal length. It can be seen from Eq. (1) that the distance between zone boundaries (${r}_{n+1}-\,{r}_{n}$) monotonically decreases with increasing $n$; hence, the fabricated minimum line width determines the outer radius limit of FZP lenses for a certain focal length, or equivalently, the maximum NA. Furthermore, it can be shown that this minimum line width is almost the same as the focused spot diameter of the FZP lens (see Supplementary Note 1 for further details). In other words, it is important to reduce the minimum line width in order to increase NA and reduce the focused beam spot size. Note that the minimum line width achievable with photolithography is a few hundred nanometers; therefore, the aspect ratio of sub-micrometer thickness thin-film structures patterned by photolithography would be limited to around 1 or less in most cases.

**Fig. 1:**
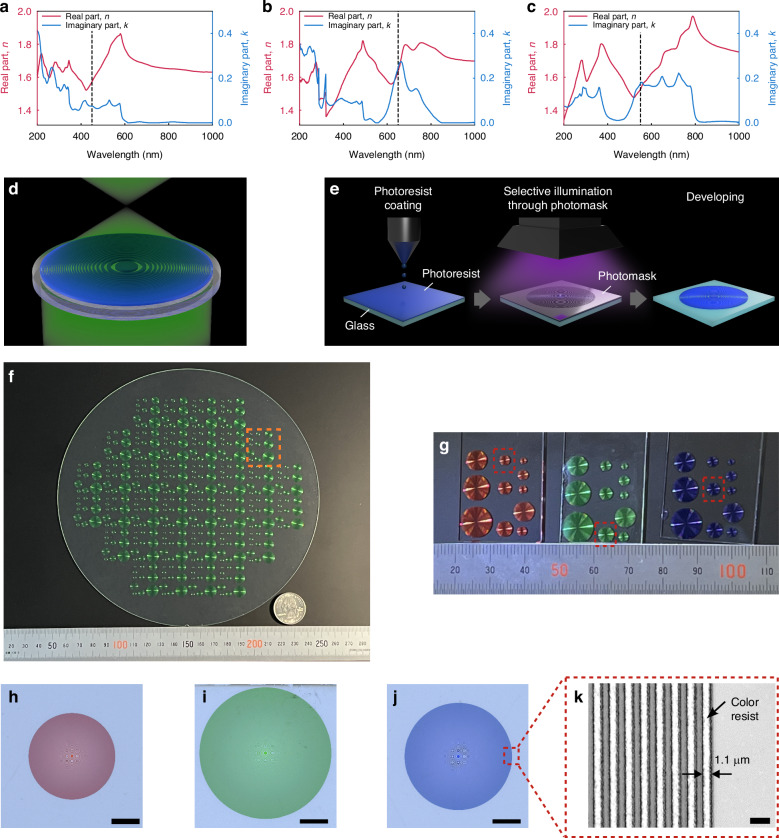
Details of FZP lens fabrication method and fabricated FZP lenses. **a**–**c** Complex refractive index spectra of red, green, and blue color resist, respectively. The dashed-vertical lines denote the design wavelength of FZP lenses fabricated with each photoresist. **d** Conceptual diagram of light focusing by FZP lens fabricated with color resist. **e** Conceptual diagram of FZP lens fabrication method. **f** Photograph of an FZP lens fabricated on an 8-inch glass substrate using green resist. **g** Photograph of the FZP lenses used in the experiment. The FZP lenses circled by the red boxes are the ones evaluated in this study. The FZP lens fabricated with green resist corresponds to the area surrounded by the orange box in (**f**). **h**–**j** Optical microscope images of the FZP lenses framed in red in (**g**). The scale bar is 1 mm. **k** Laser confocal microscope image of the area circled by the red frame. Opaque (white in the image) resist-coated areas and transparent (dark gray) non-coated areas alternate, with the outermost line having a width of 1.1 µm. The scale bar is 2 µm

For the opaque material constituting the FZP, we explored the use of color resist, a special type of photoresist that absorbs light at certain wavelengths. Three color resists with different transmission spectra were employed, henceforth referred to as red (RED-101), green (JSSG-9135), and blue (BLUE-105) photoresist, all from JSR Corporation. The complex refractive index of each material measured by spectroscopic ellipsometry (M-2000D, J. A. Wollam) is shown in Fig. 1a–c. The red photoresist can be seen to be opaque at 200 to 580 nm, the green at 200 to 480 nm as well as 610 to 810 nm, and the blue at 200 to 370 nm as well as 480 to 800 nm. An amplitude-type FZP can be designed to operate at any one wavelength within the opaque range of the respective resist. As a demonstration, we create FZP lenses working at red (650 nm), blue (450 nm), and green wavelengths (550 nm) with the red photoresist as the blue-opaque material, green photoresist as the red-opaque material, and the blue photoresist as the green-opaque material. The design wavelengths selected for each color resist are shown by the dashed-vertical line in Fig. 1a–c.

An illustration of an FZP lens fabricated using color resist as proposed in this study is shown in Fig. 1d. By curing the color resist in an FZP pattern onto a transparent substrate, lens functionality should be realizable. The FZP-lens fabrication procedure is depicted in Fig. 1e. Color resist is spin-coated onto a glass substrate surface, after which a mask pattern is exposed via a conventional semiconductor photolithography equipment (stepper) and subsequently developed. More details on the sample fabrication recipe and specific conditions are given in the “Sample fabrication” part of the “Materials and methods” section. We note that as the structure consists of only photoresist material, the fabrication procedure is greatly simplified compared to conventional metasurface fabrication schemes.

Figure 1f shows an example of the results of FZP lens fabrication on the entire surface of an 8-inch glass substrate using green color resist. The whole surface of the 8-inch wafer is covered with numerous FZP patterns of different radii and focal lengths. The use of steppers, commonly used in semiconductor manufacturing, makes it possible to apply this technology to such large-area wafers. A portion of the wafer, as shown in the dashed-orange box in Fig. 1f, cut out with a glass cutter, is shown in the middle of Fig. 1g. Similarly, FZP lenses fabricated with red and blue resist are also shown in Fig. 1g. The lenses whose characteristics were evaluated in this paper are marked by the dashed-red box in Fig. 1g. The corresponding optical microscope (VHK-7000, Keyence) images of these lenses are shown in Fig. 1h–j. Each lens was designed with a focal length of 5 mm for its respective wavelengths, and the lens diameters are 3.0 mm (red resist), 4.5 mm (green resist), and 3.7 mm (blue resist). The respective thicknesses are 0.49 μm (blue resist), 0.51 μm (green resist), and 0.47 μm (red resist) (see Supplementary Note 8). Based on Fresnel’s formulas and refractive index and extinction coefficient described in Fig. 1a–c, 87%, 92%, and 64% of the incident light is blocked by the resist at the opaque zones. While there is a little leakage, they show good light-focusing characteristics, as shown in the subsequent sections. The lens diameters were chosen so that the design outermost line width of the resist pattern for all lenses were the same at 0.8 μm, which is of the order of the fabrication resolution of the current process. A laser confocal microscope (VK-X1000, Keyence) image of the outermost region of the blue resist FZP lens (Fig. 1j) is shown in Fig. 1k. The fabricated resist patterns were slightly larger at the edges, at 1.1 μm compared to the design of 0.8 μm. The possible fabrication resolution achievable with our current setup and method are described in more detail in Supplementary Note 9. The effect of this difference on overall lens performance is discussed in subsequent sections.

### Focusing characteristics of FZP lenses

**Fig. 2:**
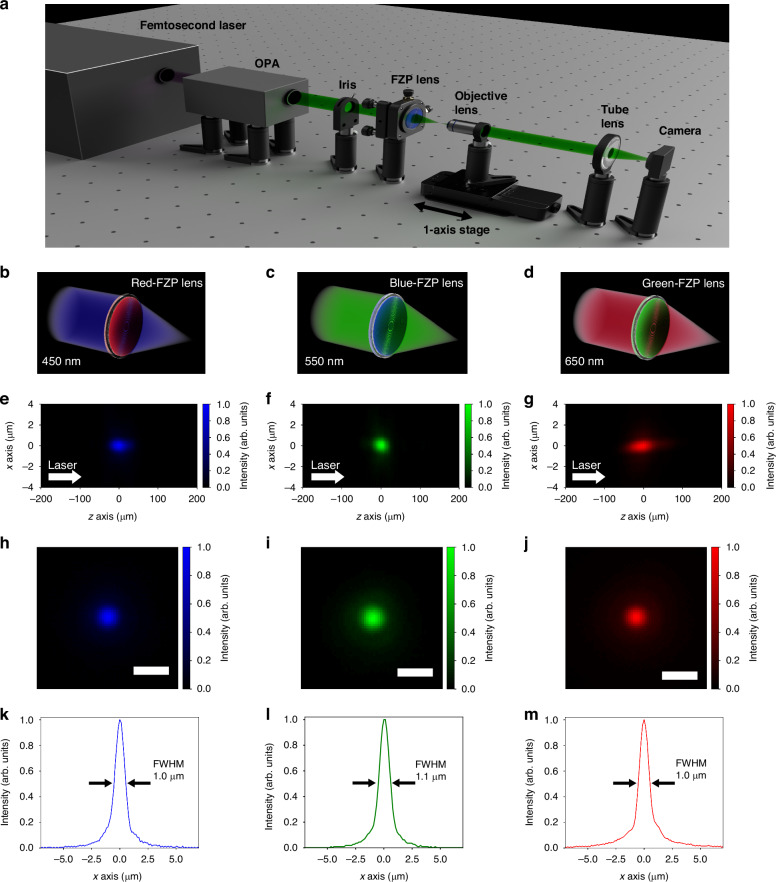
Focusing profile measurement system of the fabricated FZP lens and experimental results. **a** Schematic diagram of focusing profile measurement system. **b**–**d** Relationship between the incident wavelength and the color resist of the FZP lens. **e**–**g** Focusing profile in the beam propagation direction. **h**–**j** Intensity profile at the focal plane. The scale bar is 2 µm. **k**–**m** Horizontal cross section of (**h**–**j**) at the peak position

The focusing characteristics of the fabricated FZP lenses (Fig. 1h–j) were evaluated by a microscope imaging system based on a tunable light source. A schematic diagram of the experimental setup is shown in Fig. 2a. Each of the color resist FZP lenses was illuminated by light centered on its respective design wavelength (${\lambda }_{d})$: the red-resist FZP lens by 450 nm light (Fig. 2b)), the blue-resist FZP lens by 550 nm light (Fig. 2c), and the green-resist FZP lens by 650 nm light (Fig. 2d). For more information on the experimental setup, see “Materials and methods”.

The imaged beam propagations for the three FZP lenses when illuminated by 450, 550, and 650 nm light are shown in Fig. 2e–g for each of the red-, blue-, and green-resist FZP lenses, respectively. z = 0 μm marks the position where the peak laser intensity was largest. By measuring the distance from this z = 0 point to the position where the image of the lens surface is clearly observed using the beam profile measurement system described above, it was verified that the beam was focused at a position 5 mm from the lens surface, as designed. Additionally, the beam profile at the z = 0 plane is shown in Fig. 2h–j. From these images, the laser spot can be seen to be spatially well-focused in both in- and out-of-plane directions. The beam profile through the center of the focus spot along the horizontal axis of Fig. 2h–j is shown in Fig. 2k–m. From this profile, the full-width at half-maximum (FWHM) of each of the spots was measured as 1.0 μm (red-resist FZP with 450 nm light), 1.1 μm (blue-resist FZP with 550 nm light), and 1.0 μm (green-resist FZP with 650 nm light). Despite the simple fabrication procedure of the presented method, these results demonstrate that each type of color-resist FZP lens is more than capable of focusing light down to approximately 1 μm, a size which typically can only be achieved by utilizing objective lenses with conventional refractive optics. It should be noted that the FWHM of the blue-resist FZP lens did not change when the incident power was increased to 3 mW. This indicates that the FZP can withstand the incidence of Class 2 laser light and will not break down in normal applications such as imaging.

To qualitatively evaluate the performance of the color-resist FZP lens, we utilized finite-difference time-domain (FDTD) numerical simulations of beam focusing using commercial numerical calculation software (Ansys Lumerical FDTD). We created a corresponding simulation model of the fabricated blue-resist FZP lens and conducted three-dimensional calculations of beam propagation of 550 nm light through this structure. To mimic the design and measured line width discrepancies at the outermost regions of the lens, the line width of the simulation model FZP lens was limited to 1.1 μm in regions where the design line width was smaller than 1.1 μm (the details of these simulation conditions are detailed in Supplementary Note 2). We also took into account the finite bandwidth of the OPA light source used in our experiment during analysis (the details of this procedure are detailed in Supplementary Note 3).

**Fig. 3:**
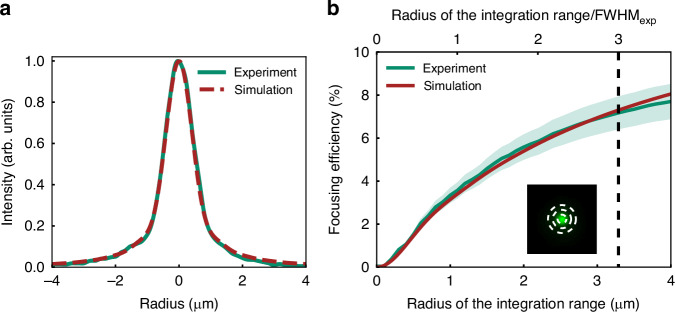
Comparison of experimental (green line) and simulated (red line) results for the FZP lens at the design wavelength of 550 nm. **a** Comparison of the intensity profiles at focus. **b** Evaluated focusing efficiency as a function of the radius of the integration range in the focal plane. The focusing efficiency was obtained by varying the signal integration area around the beam peak, as shown by the white dotted line in the inset figure for three different integration radii. The lower horizontal axis shows the radius of the integration range, and the upper horizontal axis shows the radius of the integration range divided by the experimentally determined FWHM value (FWHM_exp_ = 1.1 μm). The light green area in the figure indicates the measurement error estimated from the standard deviation of the laser beam power measured with a power meter

The calculated results are shown in Fig. 3a. Here, the beam profile at the focus is plotted by averaging the horizontal and vertical cross-sections of the measured or calculated beam profiles along the beam center. The green line denotes the experimental profile, while the red-dashed line is the simulation result. Near-perfect agreement can be observed between experiment and simulation. The FWHM determined here was 1.1 μm, in agreement with the experimental result. This result shows that the focusing properties of the FZP lenses can be well-reproduced by numerical simulations.

### Focusing efficiency measurements

Next, we quantitatively evaluated the focusing efficiency of the blue-resist FZP lens operating at 550 nm. In general, the focusing efficiency is calculated by dividing the amount of power incident around an area near the focal point at the lens focal plane by the total laser power incident upon the lens *E*_0_. While determining this value, there is some ambiguity regarding the size of the area to summate over around the focal point^33^. While one standard often seen in the literature is measuring the power within a region of radius three times the FWHM of the focused beam^8,11^, to provide a more complete analysis, we calculate the encircled power $E\left(R\right)\equiv 2 \pi {\int }_{0}^{R}I(r){rdr}$. $I(r)$ is the incident laser power at a distance of $r$ from the lens axis, or experimentally, the beam peak, and $R$ is the radius to which the incident power is integrated over^33^. The focusing efficiency for a given $R$ is then determined as $E(R)/{E}_{0}$, while choosing $R=3\times \text{FWHM}$ is equivalent to the traditional metric.

Figure 3b shows the result of this analysis. The green line is the experimental result, where energy-calibrated pixel counts are integrated numerically from the obtained beam profile at the focal point. For example, the white-dashed lines in the inset image in Fig. 3b show integration areas of 1, 2, and 3 times the FWHM. The green-filled region corresponds to the measurement uncertainty originating from the power meter. The full specifics of the measurement and calibration procedure are provided in Supplementary Note 5. According to the experimental results, when ${\text{FWHM}}_{\exp }$ is set to 1.1 μm, the focusing efficiency obtained experimentally for ${3\times \text{FWHM}}_{\exp }$ was 7.2$\pm$0.8%, as shown by the black-dashed vertical line of Fig. 3b. The same analysis conducted for the simulation results shown in Fig. 3a, which considers the spectral width of the pulse, represented by the red line in Fig. 3b. The experimentally and numerically obtained focusing efficiency results show very good quantitative agreement across the entire range of integration radii. The focusing efficiency to ${3\times \text{FWHM}}_{\exp }$ was calculated to be 7.3%, which is in complete agreement with the experimental results within measurement uncertainty.

From these results, it can be concluded that the experiments and simulations agree quantitatively. This paves the way for fully quantitative design and optimization of the color-resist FZP lenses.

### FZP lens performance when using a monochromatic laser source

In this study, we demonstrated a simple method to fabricate lenses for the visible range with focusing capabilities comparable to conventional objective lenses. While the findings agree with numerical simulation results, when considering features predicted from typical textbook results of FZP lenses (see Supplementary Note 1), the performance is slightly less than ideal. One factor is the fact that textbook results postulate plane-wave illumination while the experiments here use Gaussian waves. We find that some differences in the spot size could be explained from this (see Supplementary Note 4). Besides this point, two other factors can be identified in our experiment that contributed to such results: the non-ideal outer line width of the resist pattern in our fabricated structure and the polychromatic nature of the ultrashort pulsed light source used to evaluate the lens performance. To better understand the effects of these factors to the observed results, we compared the simulation results of ideal and fabricated blue-resist FZP lenses irradiated by both monochromatic and polychromatic light around the design wavelength of 550 nm.

**Fig. 4:**
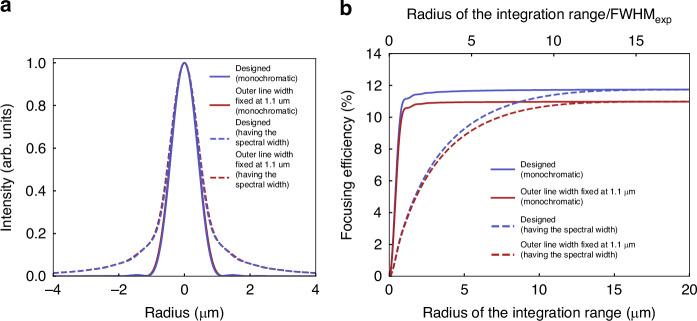
Simulation results comparing the focusing of a monochromatic light source with the focusing of a light source with a spectral width of 10 nm for the FZP lens at the design wavelength of 550 nm. The solid line represents the results of monochromatic results and the dashed line represents the results of an analysis that considers spectral width. The purple line shows the simulation results for the FZP lens model with a minimum line width of 0.8 µm as designed, and the red line shows the simulation results for the FZP lens model reflecting the actual structure (line width smaller than 1.1 µm in the design are fixed at 1.1 µm). **a** Comparison of light intensity profiles at the focus. **b** Evaluated focusing efficiency as a function of the radius of the integration range in the focal plane. The lower horizontal axis shows the radius of the integration range, and the upper horizontal axis shows the radius of the integration range divided by the experimentally determined FWHM value (FWHM_exp_ = 1.1 μm)

The calculated results for both the monochromatic and polychromatic cases for the two structures are shown in Fig. 4. The blue traces are for the ideal structure, while the red traces are for fabricated structures with minimal line widths capped at 1.1 μm; the solid lines correspond to illumination with monochromatic 550 nm light, while the dashed line corresponds to irradiation by light centered at 550 nm with a bandwidth of 10 nm. Figure 4a compares the observed beam profiles of the four cases at the focal point. As can be seen from the overlapped red and blue lines, deterioration in the line width resulted in little appreciable change; for example, the FWHM of the monochromatic beam profile was almost unchanged at 0.91 µm for the ideal structure and 0.94 μm for fabricated structures. In contrast, there are notable changes due to chromatic effects, as seen by the non-overlapped dashed and solid lines. The FWHM were slightly increased from 0.91 μm to 1.02 μm for the ideal structure and 0.94 µm to 1.06 μm for fabricated structures. More prominently, the wings of the beam profile are notably larger for the polychromatic case. We consider that this mainly reflects the fact that different colored light is focused at different focal distances, resulting in defocused contributions to the beam profile, as detailed in Supplementary Note 3.

Next, in Fig. 4b, the focusing efficiencies of the four cases are shown. In all cases, the focusing efficiencies monotonically increase, until asymptotically reaching a saturated value for a large enough integration area. In the case of the monochromatic illumination, the focusing efficiencies saturate quickly at an integration radius of about two times the FWHM. In contrast, for the case of polychromatic illumination, the focusing efficiency saturates much slower: they do not reach an asymptotic value until close to 14 times the FWHM radius. However, the asymptotic saturated values themselves are not affected by the illumination method and are influenced only by the minimum line width, having a final value of 11.7% and 11.0% for the designed and fabricated structure, respectively.

From the above results, it becomes apparent that the beam radius of the focal spot is greatly influenced by the bandwidth of the light used to evaluate the FZP lenses. Conversely, the discrepancy between the fabricated structure line widths and the designed line widths contributes to a decrease in the absolute focusing efficiency. Both factors affected the final evaluated performance of the presented FZP lenses. However, even with the fabricated FZP lens, provided a more favorable evaluation with monochromatic light, Fig. 4b reveals that the nominal focusing efficiency should be improved from 7.2% to close to 10.9% (at 3 times the FWHM).

### Imaging demonstration

**Fig. 5:**
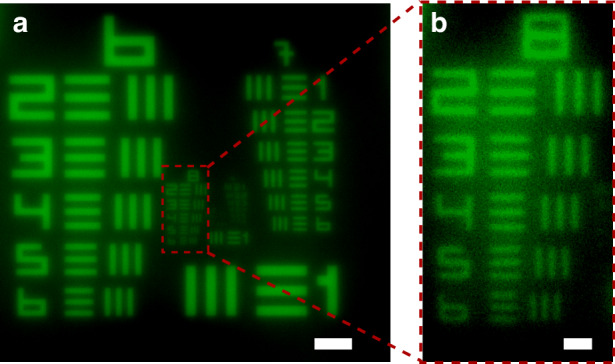
Imaging results of a USAF 1951 resolution target using the FZP lens designed for a wavelength of 550 nm. **a** Group 6, 7 and 8 imaging results. Scale bar size is 30 µm. **b** An enlarged image of the Group 8 area enclosed by the red dashed line in (**a**). The size of the scale bar is 5 µm

Lastly, we evaluated the imaging capabilities of our fabricated FZP lens (the measurement setup is shown in Supplementary Note 6). We utilized a Negative-type 1951 United States Air Force (USAF) resolution test chart (HIGHRES-1, Newport) as the imaging target. The imaging results with the color-resist FZP lens are shown in Fig. 5. As can be seen in Fig. 5a, the elements up to group 7 in the test chart can be clearly recognized. Upon closer inspection, an enlarged image of the group 8 region in Fig. 5a is shown in Fig. 5b. Here, the image is apparently able to resolve element 6 in group 8, which has a line width of just 1.1 μm. From these measurements, it is evident that our fabricated FZP lenses are effective for imaging applications.

## Discussion

From the introduced results, it is apparent that our fabricated FZP lens are useful for visible light focusing and imaging. A comparison of our lens performance with representative works in the literature is provided in Table 1. The experimental spot size for our lens is smaller than that reported for other FZP type lenses. Regarding the focusing efficiency, while it is still lower than values for other metalens types, it still shows values comparable to other amplitude-type FZP lenses. We note that if we consider the predicted monochromatic focusing efficiency calculated from simulations (10.9%), our result would be the most efficient among the amplitude-type FZP lenses. Whereas resist material was used as an optical mask to create amplitude-type FZP lenses, if we were to instead create designs to utilizing the transparent region of resist material to create phase-type FZP lenses, the focusing efficiency could theoretically become up to four times higher^34^. Such efforts would require the precise control of photoresist thickness in addition to lateral dimensions.

**Table 1:** Summary of planar lens types, materials, fabrication techniques, design wavelength, diameter, focal length, numerical aperture (NA), FWHM, and focusing efficiency for this and previous studies reported in the literature

Ref.	Type	Material (substrate)	Method	Wave-length (nm)	Diameter (mm)	Focal length (mm)	NA	FWHM (µm)	Focusing Efficiency (%)
This work	FZP (Amplitude type)	Color resist (glass)	Photo Lithography	550	3.659	5	0.37	1.1	7.2^※5^
^21^	FZP (Amplitude type)	Ag/Au/Al (SiO_2_)	EB lithography, PVCVD × 3	450 /550 /650	0.210	1	0.11	2.60 /2.43 /2.11	5.8 ~ 8.7
^20^	FZP (Amplitude type)	Cr (SiO_2_)	EB lithography	635	0.500	2	0.13	2.88	10
^30^	FZP (Amplitude type)	Au (SiO_2_)	Laser processing	633	1.016	12	0.04	10	4.17
^35^	FZP (Phase type)	Polymer	light-induced structure	633	0.190	0.5	0.19	1.7	34
^20^	MZP^※1^	A-Si:H (SiO_2_)	EB lithography	635	0.500	2	0.13	2.8	14.6
^36^	FZP	SiO_2_	DUV Lithography, ICP etching, reactive ion etching (RIE)	405	4.13	1	0.9	0.17 ~ 0.21	13.8
^37^	Photon sieves	MoS_2_	PVD, FIB etching, Chemical etching	450	0.08	0.02	~0.9	0.198	22
^38^	supercritical lens	Cr (SiO_2_)	UV laser lithography, Etching	405	0.59	0.055	-	0.165	-
^11^	Metalens	TiO_2_ (SiO_2_)	EB lithography, RIE	650-1000	0.030	0.06	0.24	~ 1.6^※2^	65^※2^
^15^	Metalens	TiO_2_ (SiO_2_)	EB lithography, Atomic layer deposition	405 /532 /660	0.240	0.09	0.8	0.280 /0.375 /0.450	86/73/66
^17^	Metalens	TiO_2_ (SiO_2_)	EB lithography, Atomic layer deposition	470-670	0.020	0.049	0.2	1.4^※3^	20^※4^
^24^	Metalens	UV-curable photoresist	UV nanoimprint lithography	532	0.5	0.5	0.45	0.8	20
^29^	Metalens	SiO_2_	DUV lithography	633	10	50	0.1	3.23	45.6

^※1^Abbreviation for Metasurface Zone Plate

^※2^Measurement results at 650 nm

^※3^Measurement results at 530 nm

^※4^Measurement result at 500 nm

^※5^Evaluated using a pulsed laser light source with a spectral width of 10 nm

In addition to the evaluation of the FZP lens at the design wavelengths, we also measure their focusing properties at other wavelengths to gauge their more general utility. Using the blue FZP lens designed for 550 nm light, we tested the focusing ability of both 450 and 650 nm light. We found that the FWHM of both were 1.2 μm, and while they were slightly larger than the spot for the design wavelength, they still operated at close to a 1 μm spot size. More details are provided in Supplementary Note 7. In addition, we tested the imaging performance of this lens for 450 nm and 650 nm light. We find here that again, while performance is degraded compared to the design wavelength, satisfactory imaging performance could still be observed, as shown in detail in Supplementary Note 7. These results show that the fabricated FZP lenses operate reasonably well even outside their design wavelengths.

In conclusion, we demonstrated a simple and large-area fabrication technique for flat FZP lenses utilizing color resist, a special type of photoresist that absorbs at certain wavelengths. We fabricated three FZP lenses designed for focusing of 450, 550, and 650 nm light, and evaluated their focusing properties with a wavelength-tunable pulse-laser system. We found that they were all able to focus the laser spot down to a diameter of approximately 1 micrometer. We evaluated the focusing efficiency of the FZP lens for 550 nm, and found an experimental value of 7.2%, a value comparable with other amplitude-type FZP lenses reported in the literature. These results were compared with numerical simulations, showing excellent agreement and demonstrating the high potential designability of our fabrication scheme. We also demonstrate that the lenses were able to image features down to 1.1 μm, which is potentially useful for imaging applications. These results are important steps towards the realization of practical and cost effective flat-lens fabrication production capable of meeting mass-production needs.

## Materials and methods

### Experimental setup for evaluation of FZP lens

The light source was a regenerative amplifier laser system (PHAROS-PH1-SP-1mJ, Light Conversion) with a pulse duration of 190 fs and a central wavelength of 1030 nm. This laser pumped an optical parametric amplifier (OPA) (ORPHEUS-HE, Light Conversion), allowing for wavelength conversion that covers the entire visible spectrum. A spatial filter (omitted from the figures) consisting of two lenses and a 10 μm diameter pinhole was used to clean the beam spatial mode profile and adjust the beam spot size to be comparable to the FZP lens diameter. An iris was used to cut the incident beam to be approximately equivalent to each lense radius. After the FZP lens, an NA 0.65 lens (PLN40X, Olympus) combined with an f = 180 mm tube lens (TTL-180A, Thorlabs) was used to magnify the focused beam of the FZP lens, which was then recorded by a CMOS camera (DCC1545M, Thorlabs). Here, the objective lens was mounted onto a one-axis automated translation stage (OSMS20-35, OptoSigma); by moving the lens, beam profile images at different planes along the beam propagation axis could be obtained. Unless otherwise noted, we show beam propagation data with the objective scanned at 2 μm increments.

### Sample fabrication

An adhesive solution (Dow Chemical Company, AP3000) was spin coated onto a glass substrate (Corning Incorporated, Eagle XG, 8-inch diameter, 0.7 mm thickness) at 1500 rpm for 23 s using spin coating equipment (Tokyo Electron Ltd., CLEAN TRACK ACT8). The substrate was heated on a hot plate at 190 °C for 300 s. Similarly, three pieces were fabricated. Green resist (JSR Corporation, JSSG-9135) was spin coated onto a glass substrate with adhesive layer at 1030 rpm for 23 s. Blue resist (JSR Corporation, BLUE-105) was spin coated onto another glass substrate with adhesive layer at 530 rpm for 23 sec. Red resist (JSR Corporation, RED-101) was spin coated onto another glass substrate with adhesive layer at 1590 rpm for 23 s. The three substrates were then baked at 100 °C for 180 s. UV irradiation was performed onto the resist coated glass substrates using an i-line stepper (Canon Inc., FPA-3030i5+) at 40 mJ/cm^2^ for red and blue resist, 70 mJ/cm^2^ for green resist, with different Cr photomasks according to each design. The irradiated UV wavelength was 365 nm. After UV irradiation, the substrates were developed using a developer (KANTO CHEMISTRY Co., Inc., S-170809, tetramethyl ammonium hydroxide solution). After rinsing off the resist developer with deionized water, post-baking was performed on a hot plate at 200 °C for 300 s. Each fabricated FZPs could then be used for optical characterization without any other treatment.

## Supplementary information

Supplemental Material

## Data Availability

The data provided in the manuscript is available from K.K. upon request.
