# Supplementary material for: Optical Fresnel zone plate flat lenses made entirely of colored photoresist through an i-line stepper
Source: Light Sci Appl. 2025 Jan 16;14:43. doi: 10.1038/s41377-024-01725-6 (PMC11735980; doi:10.1038/s41377-024-01725-6)
Supplement: Supplementary file 1 — Supplemental Material [file 41377_2024_1725_MOESM1_ESM.pdf]

Supplementary Information for
Optical Fresnel zone plate flat lenses made entirely of colored photoresist through an i-line stepper

Ryohei Yamada[1], Hiroyuki Kishida[2], Tomohiro Takami[2], Itti Rittaporn[2,3], Mizuho Matoba[1], Haruyuki Sakurai[1], Kuniaki Konishi[1*]

1. Institute for Photon Science and Technology,
The University of Tokyo, Tokyo, 113-0033, Japan
2. JSR Corporation, Tokyo, 105-8640, Japan
3. JSR-UTokyo Collaboration Hub, CURIE, Tokyo, 113-0033, Japan

e-mail: kkonishi@ipst.s.u-tokyo.ac.jp

Supplementary Note 1: Relationship between the minimum line width of a FZP lens and the FWHM of the intensity profile at the focus

In previous studies, the relationship between the minimum line width Δr of a FZP lens and the FWHM of the beam profile of a beam focused by this lens is given as both FWHM $= 1.22\Delta r$ [1-4] and FWHM $= \Delta r$ [5], We use the latter approximation in the current study. To justify this approximation, we re-derive the relationship between the FWHM and Δr here analytically.

From equation (1) of the FZP lens in the main text, the focal length f, the wavelength λ, and the radius of the ring when the outermost ring is the n-th ring can be written as follows:

$$r_n^2 = n\lambda f + \frac{n^2\lambda^2}{4} \ .(S1)$$

The line width of the outermost opaque region is then defined as follows:

$$\Delta r \equiv r_n - r_{n-1} \ .(S2)$$

Calculating the difference between the squares of the radii of the n-th and (n-1)-th rings yields

$$r_n^2 - r_{n-1}^2 = \left(n\lambda f + \frac{n^2\lambda^2}{4}\right) - \left\{(n-1)\lambda f + \frac{(n-1)^2\lambda^2}{4}\right\} = \lambda f + \frac{(2n-1)}{4}\lambda^2 \ . \ (S3)$$

Using Δr in equation (S1), the left-hand side of equation (S3) can be written as

$$r_n^2 - (r_n - \Delta r)^2 = 2r_n\Delta r - (\Delta r)^2 \simeq 2r_n\Delta r \ . \ (S4)$$

Here, we used $\Delta r \ll r$ when n is sufficiently large. Equations (S3) and (S4) lead to

$$\lambda f + \frac{(2n-1)}{4}\lambda^2 \simeq 2r_n\Delta r \ . \ (S5)$$

Let the diameter of the FZP lens be $D = 2r_n$, and rearranging the above equation for f yields

$$f \simeq \frac{D\Delta r}{\lambda} - \frac{2n-1}{4}\lambda. \ (S6)$$

The numerical aperture (NA) of the FZP lens is defined as follows:

$$\mathrm{NA} \equiv \sin\theta, \ (S7)$$

where θ is the half angle of the cone created by the lens focal point and the lens. Since NA $= r_N/f = D/2f$, using equation (S6), the NA of the FZP lens is

$$\mathrm{NA} \simeq \left[2\left(\frac{\Delta r}{\lambda} - \frac{2n-1}{4D}\lambda\right)\right]^{-1} \ . \ (S8)$$

When a plane wave enters the FZP lens, the intensity profile at the focus of the primary diffracted light is an airy pattern, and the radius R_0 at which the intensity first becomes zero can be written using the numerical aperture as follows [6]:

$$R_0 = \frac{0.610\lambda}{\mathrm{NA}} \ . \ (S9)$$

Substituting equation (S8) into equation (S9) yields

$$R_0 \simeq 1.220 \times \left(\Delta r - \frac{2n-1}{4D}\lambda^2\right) \ .(S10)$$

In order to clarify the relationship between FWHM and minimum line width from the above equation, we obtain the relationship between the radius $R_{1/2}$, defined as the radius where the intensity is 1/2 of the maximum, and the radius R_0. First, as the intensity profile at the focus is an Airy pattern, it can be represented by the following Airy function

$$I(R) = I_0 \left|\frac{2J_1(R)}{R}\right|^2, \ (S11)$$

where I_0 is the peak intensity and $J_1(R)$ is the Bessel function of the first kind. From equation (S11), the values of $R_{1/2}$ and R_0 are 1.616 and 3.832, respectively, and the ratio of the two is $R_{1/2}/R_0$=1.616/3.832=0.4218 (see Fig. S1). Therefore, $R_{1/2}$ can be written in terms of Δr using equation (S10) as follows:

$$R_{1/2} = 0.4218 \times R_0 \simeq 0.5146 \times \left(\Delta r - \frac{2n-1}{4D}\lambda^2\right) \ . \ (S12)$$

From equation (S12), the FWHM becomes

$$\mathrm{FWHM} = 2 \times R_{1/2} \simeq 1.029 \times \left(\Delta r - \frac{2n-1}{4D}\lambda^2\right) \quad .(S13)$$

In the case of the FZP lens fabricated in this study with blue resist, the design wavelength is 550 nm, the focal length is 5 mm, the number of rings is 1179 with a corresponding diameter of 3.7 mm, and the line width of the outermost opaque region is 0.8 µm. Here, $(2n-1)\lambda^2/(4D)$=0.049 µm, and consequently $(2n-1)\lambda^2/(4D) \ll \Delta r$. Therefore, equation (S13) can be further approximated as:

$$\mathrm{FWHM} \simeq \Delta r. \quad (S14)$$

The above shows that the FWHM of the intensity profile at the focus is comparable to the minimum line width Δr. The above results are for the case of a plane wave in the FZP lens, but simulations have shown that the value of FWHM is slightly larger for Gaussian beams. For more details, see Supplementary Note 4.

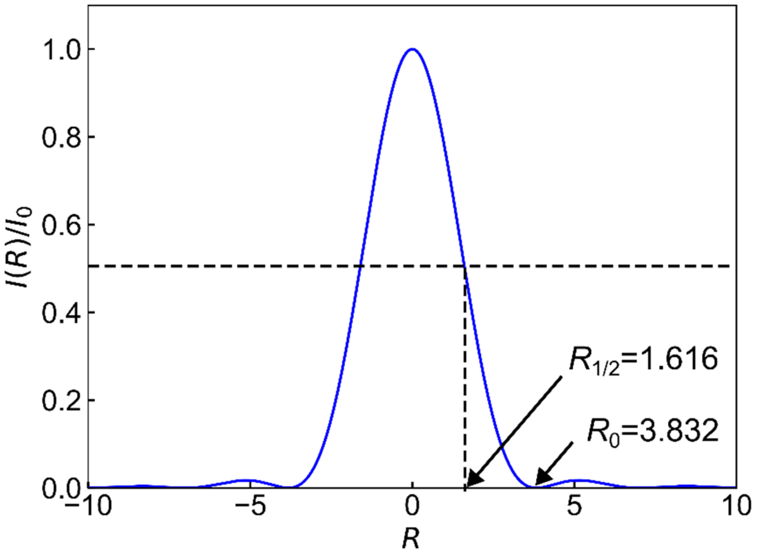

Fig. S1. Graph showing the radius at which the intensity is half of the peak intensity in the Airy function and the radius at which it first reaches zero.

Supplementary Note 2: Focusing Simulation Methods

This section describes the numerical simulation method used to obtain the focusing profile of light passing through the FZP lens. A commercial finite difference time-domain (FDTD) solver (Ansys Lumerical) was used for the simulation. Fig. S2 shows schematic diagrams of the computational domain of the FDTD. Fig. S2a is a three-dimensional schematic diagram of the computational domain and Fig. S2b is a cross-sectional view when cut by the black dashed line in Fig. S2a. For the FZP resist pattern, the design wavelength is set to 550 nm, the focal length to 5 mm, and the diameter to 3.7 mm. To reflect the structure of the actual FZP lens fabricated, the line width of the opaque region is set according to Eq. (1) in the text until the line width of the opaque region formed by the blue resist is 1.1 μm, and outside this point, the line width of the opaque region is set constant at 1.1 μm. The thickness of the colored resist was set to 0.490 μm, which is the same as the measured thickness. The diameter of the glass was set to 3.8 mm so that the glass is present up to the FDTD boundary in the region above the colored resist. For the physical properties of the colored resist, the real and imaginary parts of the complex refractive index measured by spectroscopic ellipsometry were used. For the physical properties of the glass substrate, a refractive index (1.518 at 550 nm) measured by spectroscopic ellipsometry (M-2000DI-T, J.A. Woollam) was used.

A Gaussian beam with a central wavelength of 550 nm with an electric field of linear polarization in the x-axis direction propagating in the z-axis direction from inside the glass 1 μm above the bottom surface of the colored resist (the boundary with air) was used as the source field. The beam diameter (at which the peak intensity is $1/e^2$) was set to be the same size as that of the FZP lens. The Rayleigh length for this beam would be 19 mm, which is sufficiently long compared to the distance to the top surface of the colored resist (the boundary surface with the glass), so the beam spread within the glass substrate before reaching the top surface of the colored resist is negligible. A monitor was placed 0.5 μm below the bottom surface of the colored resist to record information on the electric field, magnetic field, and pointing vector of the light passing through the structure. The target simulation space for FDTD was -1900 to 1900 μm for the x and y directions and -1 to 1 μm for the z direction, with the center of the lower surface of the colored resist taken as the origin, with perfectly matched layer boundary conditions on all sides. In actual implementation, an anti-symmetric plane coincident with the xz plane and a symmetric plane coincident with the yz plane could be identified in the simulation space. By utilizing these features, the computational domain could be reduced by a factor of four by only calculating the first quadrant of the target simulation space in xy space. In this case, the boundary conditions are modified as shown in Tab. S1, where x_{min} and y_{min} are now the 0 position at the center of the simulation space.

The electromagnetic field information in the near field obtained from the FDTD calculation was then projected to the far field [7], allowing us to determine the electric field distribution at the focal plane of the FZP lens, and hence, the intensity distribution. The calculation range within the focal plane was -10 to 10 μm in the x and y directions, and the mesh size was 0.1 μm.

The focusing efficiency was evaluated by calculating the encircled power (see main text) around the center of the beam (x=y=0) and dividing this value by the total integrated intensity of the light incident on the FZP lens at the source position.

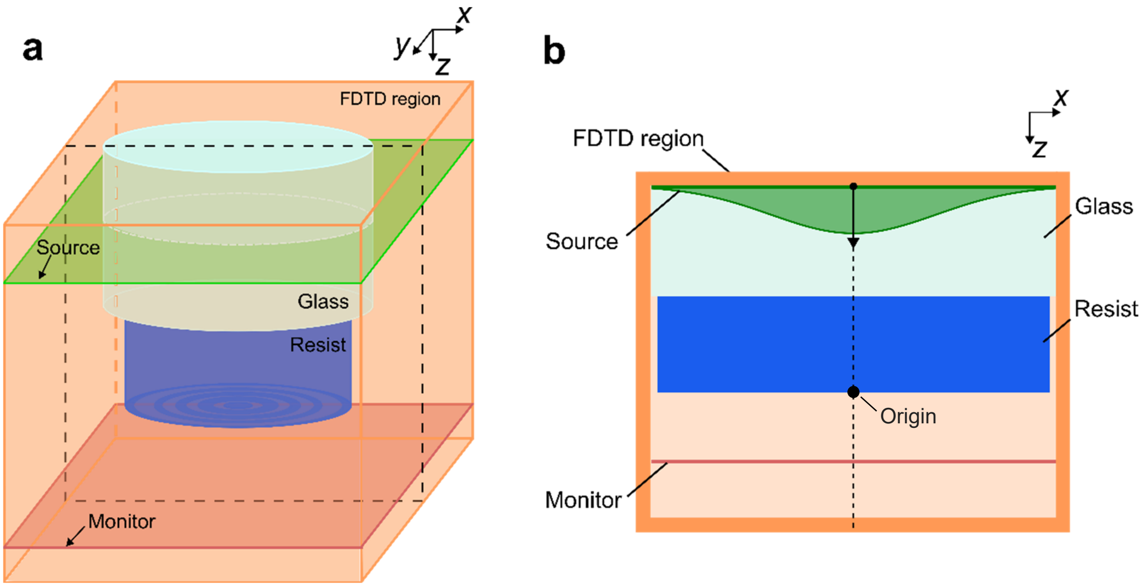

Fig. S2. Schematic diagram of the simulation space. **a**. Schematic diagram of the simulation space in three dimensions. **b**. Cross-sectional view of **a**, cut by the plane denoted by the black dashed line.

Tab. S1. Boundary conditions used in the simulation

Boundary	Boundary Condition
x_{min}	Anti-Symmetric
x_{max}	Perfectly Matched Layer
y_{min}	Symmetric
y_{max}	Perfectly Matched Layer
z_{min}	Perfectly Matched Layer
z_{max}	Perfectly Matched Layer

Supplementary Note 3: Effect of the spectral width of the light source on the simulation results

This section describes the analytical method used to account for the spectral width of the light source obtained experimentally, which should be contrasted to the monochromatic focusing profile obtained by numerical simulation. The spectrum of the ultrashort pulsed laser source used in the experiment was measured with a spectrometer (CCS200, Thorlabs), and the results are shown in Fig. S3a. The blue, green, and red lines are the spectra of the emitted light from the optical parametric amplifier (OPA) when the emission wavelength was set to 450 nm, 550 nm, and 650 nm, respectively. In particular, for the measurement results with a wavelength of 550 nm, it can be seen that the spectrum has a width of about 10 nm (Fig. S3b).

For an FZP lens with a design wavelength of 550 nm and a design focal length of 5 mm, the actual focal length of each wavelength in the experimentally measured spectrum can be determined from equation (1) in the main text as

$$f = \frac{r_n^2}{n\lambda} - \frac{n\lambda}{4}. \quad (S15)$$

Using this formula, the focal position of each wavelength component in the spectrum of the light source can be calculated. The results for the present design value of λ = 550 nm, r_n = 1.83 mm, and the minimum line width of 0.8 μm (n = 1179) are shown in Fig. S3c. It can be seen that the focus position of each wavelength is located in a range of approximately ±50 μm relative to the focal length of 5 mm at the design wavelength.

Next, Fig. S4a shows the numerical simulation results when a 550 nm monochromatic laser beam is incident on an FZP lens with a design wavelength of 550 nm and a focal length of 5 mm, which was fabricated using the blue color resist shown in the main text. Here, z=0 μm is the focal position of the FZP lens. Compared with the experimental results shown in Fig. 2f in the main text, the intensity profile has a smaller spread along the z-axis. The intensity profiles at various z positions are shown in Fig. S4b. The peak intensity can be seen to rapidly decrease as the beam spreads away from the focal point, and the formation of a pedestal can be observed in the wings of the profile. From the above points, it is expected that in the case of a light source with a broad spectrum, light with a wavelength different from the design wavelength would be focused at a position different from the focal point and hence, defocused at the design focal plane; the contribution from such light to the total intensity profile in the design focal plane would have a decreased peak intensity and a broader base area.

In order to obtain the total intensity profile at the design focal plane (z=0 μm) for a light source with a finite spectral width, we utilized the above simulation results for the 550 nm light with the following procedure:

1. The intensity profile at z=0 μm for an arbitrary wavelength was assumed to be equivalent to the defocused intensity profile of the design wavelength simulation results, where the defocusing distance was determined as the distance between the two wavelengths' focal distances calculated from equation (S15).
2. The contribution of the wavelength component was weighed by the relative intensity of the experimentally measured spectrum.
3. The total profile was determined by summing the weighted intensity profiles over the light source spectrum.

This procedure allowed us to avoid repeating the costly near-field calculations for each slightly differing wavelength. The intensity profiles at the focal plane obtained from this procedure are shown by the red dashed lines in Fig. 3a and Fig. 4a in the main text. Similarly, we applied the above procedure to calculate the focusing efficiency when the spectral width is taken into account (red line in Fig. 3b of the text).

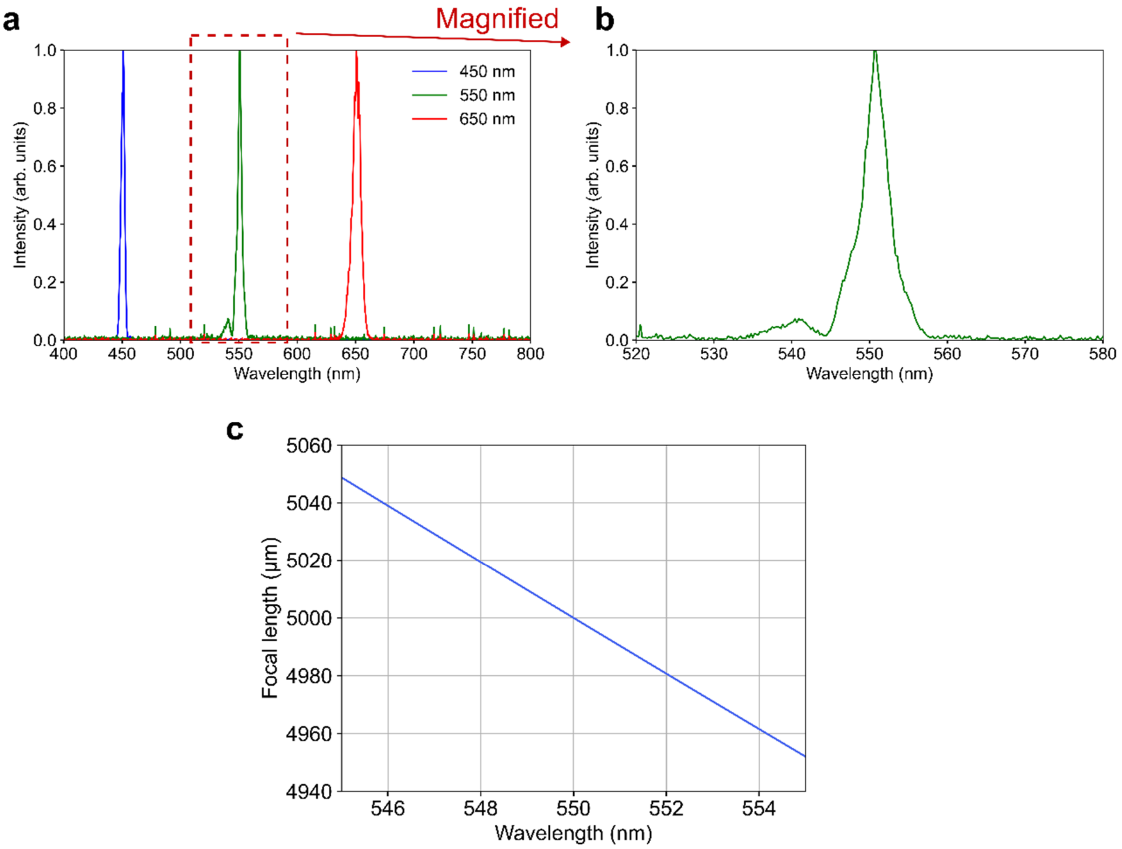

Fig. S3 Spectra of the light sources used in the experiments and the corresponding focal lengths of the FZP lenses. **a** Laser light spectrum emitted from the OPA at 450 nm (blue line), 550 nm (green line), and 650 nm (red line). **b** Magnified graph of the 550 nm spectrum in **a**. **c** Focal length of the FZP lens corresponding to the wavelength range of the light sources.

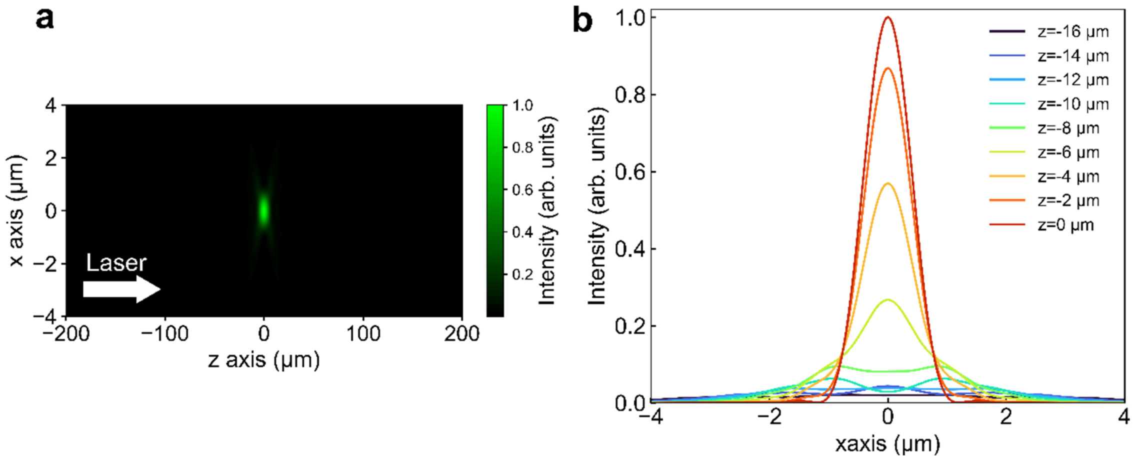

Fig. S4 Dependence of intensity profiles obtained by simulation on z-position. **a** Intensity profile in the xz plane. **b** Dependence of intensity profiles on z-position.

Supplementary Note 4: Comparison of beam profiles at the focus with plane wave and Gaussian beam in the simulation

Here we compare the intensity profiles at the focal plane for plane wave and Gaussian beam incidence on an FZP lens. Simulations were performed for an FZP lens with blue resist, design wavelength of 550 nm, focal length of 5 mm, minimum line width of 0.8 µm, and diameter of 3.7 mm. The intensity profiles at the focal plane for plane wave and Gaussian beam incidence are shown in Fig. S5 as blue and orange lines, respectively. It can be seen that the beam is more spread out when a Gaussian beam is incident than when a plane wave is incident. The FWHM values were 0.8 µm for the plane wave and 0.9 µm for the Gaussian beam. As was shown in Eq. (S14), the FWHM at the focal point is well approximated by the minimum line width of the FZP lens when plane waves are incident. On the other hand, it is slightly larger than the line width in the case of a Gaussian beam.

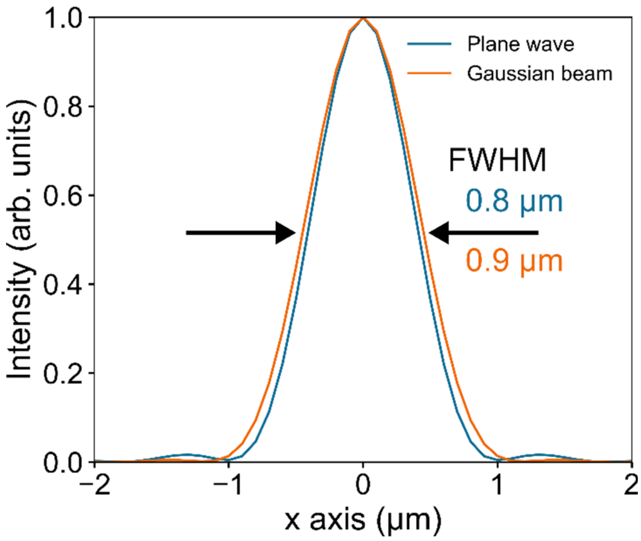

Fig. S5. Comparison of focusing profiles when plane waves (blue line) and Gaussian beams (orange line) are incident on the lens.

Supplementary Note 5: Method for measuring light focusing efficiency of FZP lenses

This section describes the method used to measure the light collection efficiency of FZP lenses. In previous studies, the focusing efficiency was measured by camera by calculating the counts at the focal plane of the lens divided by the counts of laser light incident on an aperture of the same size as the diameter of a lens [8]. This method is effective when the lens size is tens to hundreds of times larger than the spot size. However, when the diameter of the lens (3.7 mm) is approximately 3,000 times larger than the spot size (1.1 μm), as in the FZP lens fabricated in this study, the method is not suitable because the laser light incident on the lens does not all enter the camera sensor surface. Therefore, in this study, the focusing efficiency was determined by combining the intensity profile measurement by the camera and power measurement by a power meter.

A schematic diagram of the optical system used to measure the light collection efficiency is shown in Fig. S6a. The basic setup, including light source, camera, etc., is the same as described in the main text. First, the power of the laser light incident on the FZP lens was measured with a photodetector-type power meter (S130C, Thorlabs). Then, the power of the laser light was sufficiently reduced using an ND filter, and the focal intensity profile was measured at the focal plane using the aforementioned measurement system (results of the focal intensity profile measurement in the main text). For background correction, we assume a uniform background signal, and the average counts in the peripheral area of the image sensor where the laser light was not irradiated was subtracted from the entire image. The dependence of the total counts on the radial direction could then be determined by integrating the counts over different integration ranges from the center of the intensity profile.

In order to determine the focusing efficiency, it is necessary to convert the counts into the power of the laser beam. Therefore, the following method was used to calibrate the camera counts and power. First, as shown in Fig. S6b, the FZP lens was replaced with an objective lens (PLN10X, Olympus) and the power after transmission through the objective lens was measured using a power meter. The camera gain and exposure time was then set to the same time as for the focused measurement of the FZP lens, and the power of the laser beam was sufficiently reduced using an ND filter to the extent that the camera was not saturated, and the intensity profile was measured at the focal plane. The background correction procedure was the same as in the case of the FZP lens described above. The total counts were obtained by integrating the counts over a range large enough for the spot size at the focal plane. Assuming that the light focusing efficiency of the objective lens was 100%, the actual power just after the objective lens during the intensity profile measurement was determined based on the power after the objective lens transmission and the attenuation ratio of the ND filter, and the power per camera count (W $count^{-1}$) was calculated from this value and the total number of camera counts. Finally, this conversion factor was used to convert the counts calculated from the results of the intensity profile measurement of the FZP lens to power. Then, based on the power just in front of the FZP lens and the attenuation ratio of the ND filter, the actual power just in front of the FZP lens during the intensity profile measurement was determined, and the focusing efficiency was obtained by dividing the power value determined

from the camera measurement by this value.

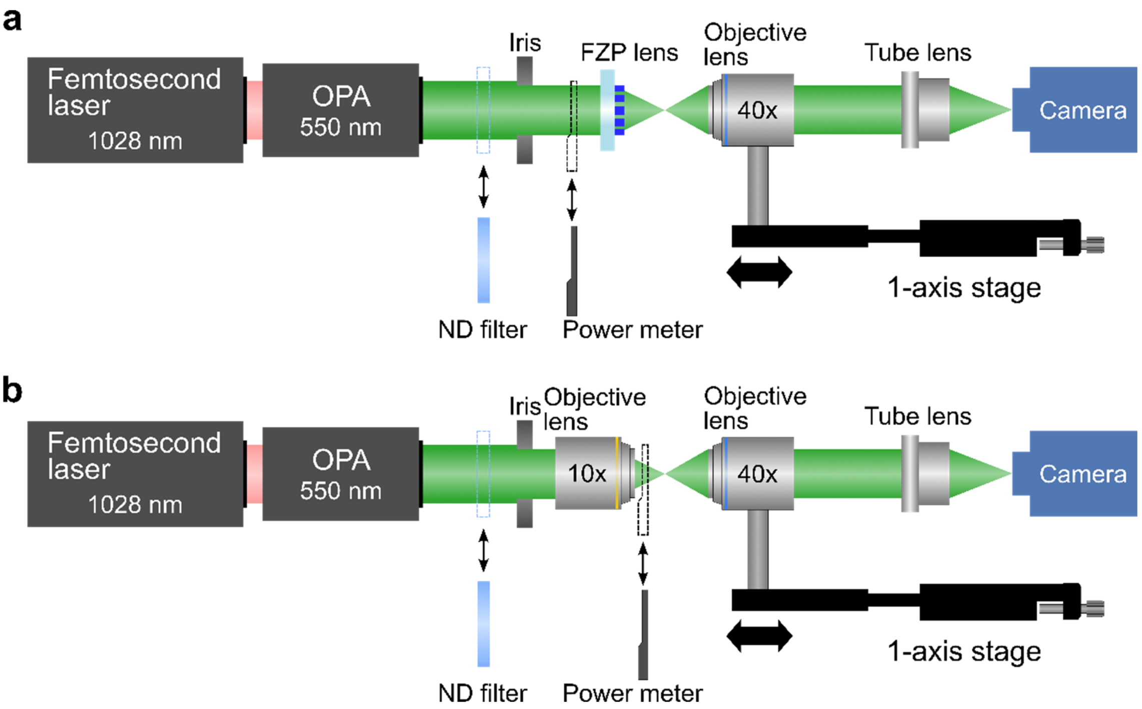

Fig. S6. Schematic diagram of the experimental system used to measure focusing efficiency. **a** For measurement of FZP lens. **b** For power calibration.

Supplementary Note 6: Imaging system for resolution targets using FZP lenses

This section describes the experimental system used for imaging the resolution target with the FZP lens at the design wavelength of 550 nm fabricated with blue resist. The measurement system used in the experiment is shown in Fig. S7. An optical microscope (VHX-7000, Keyence) was used for the measurements. A bandpass filter (FBH550-10, Thorlabs) was used to illuminate the object with monochromatic light of wavelength 550 nm from the white light source of the optical microscope. A negative-type 1951 United States Air Force (USAF) resolution test chart (HIGHRES-1, Newport) was used as the target, which was placed between the light source and the FZP lens. The FZP lens was placed at 6 mm from the resolution test chart, and the actual image magnified by a factor of 5 was observed with an optical microscope. An objective lens with a magnification of 100x was used for the measurement of groups 6 and 7 of the resolution test chart, and an objective lens with a magnification of 300x was used for the measurement of group 8.

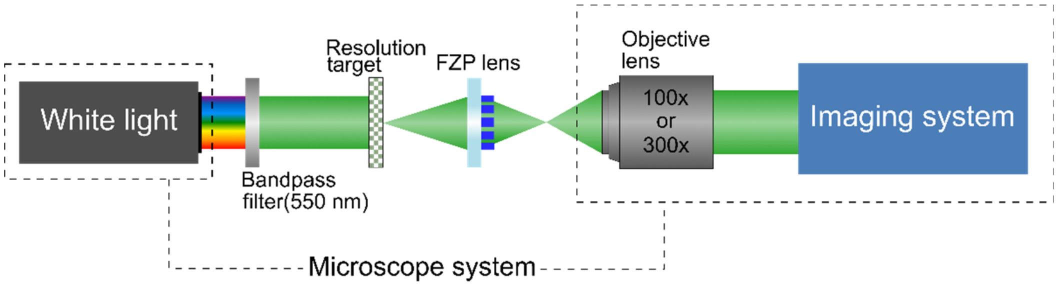

Fig. S7. Schematic diagram of the measurement system used for imaging the resolution target.

Supplementary Note 7: Evaluation of the focusing characteristics of a laser beam with a wavelength different from the design wavelength

Focusing properties were evaluated when a laser beam with a wavelength different from the design wavelength was incident on the FZP lens. Using the focusing profile measurement system shown in the main text, we measured the focusing profiles of laser beams of 450 nm, 550 nm, and 650 nm incident on an FZP lens with a design wavelength of 550 nm and a focal length of 5 mm, which was fabricated using the blue resist shown in the main text. The results are shown in Fig. S8a-c. For each wavelength, measurement results along the z-axis are shown in Fig. S8d-f, respectively (for 550 nm, the results are identical to those shown in the main text). Here, z=0 µm is the point of highest light intensity on the z-axis for each wavelength. Compared to the design wavelengths, the beams at 450 nm and 650 nm are broadened with respect to the z-axis. From equation (1) in the main text, the focal length f_{d} when the light of the design wavelength λ_{d} is incident to the FZP lens is given by the following equation:

$$f_{\mathrm{d}} = \frac{r_n^2}{n\lambda_{\mathrm{d}}} - \frac{n^2\lambda_{\mathrm{d}}}{4}. \quad (S16)$$

The focal length when light of a wavelength λ' different from the design wavelength is incident is expressed by the following equation:

$$f' = \frac{r_n^2}{n\lambda'} - \frac{n^2\lambda'}{4}. \quad (S17)$$

From (S16) and (S17), the ratio of the focal length at a wavelength different from the design wavelength (f'/f_{d}) to the focal length at the design wavelength can be obtained. In the case of r_n=1.83 mm and the minimum line width of 0.8 µm (n=1179), the above equation indicates that when the light of λ=450 nm and 650 nm enter the FZP lens, it is focused at 1.2 times and 0.84 times the focal position of 5 mm at the design wavelength, respectively. In this measurement, the laser beams of 450 nm and 650 nm were focused at 1.2 and 0.84 times the designed positions, respectively, confirming that the performance is as designed.

Next, the in-plane intensity profiles at z=0 µm are shown in Fig. S8g-i. It can be seen that the laser beam is well focused at the center of the focal plane in all cases. The cross-sectional intensity profiles of these measurements in the transverse direction are shown in Fig. S8 -l. The full-width at half-maximum (FWHM) from each intensity profile shows that the FWHMs of the 450 nm, 550 nm, and 650 nm design FZP lenses were 1.2 µm, 1.1 µm, and 1.2 µm, respectively. These results confirm that FZP lenses made with this simple method can focus light in the visible region to approximately 1 µm, even when light of wavelengths other than the design wavelengths is incident.

The measurement system shown in Supplementary Note 6 was then used to image the resolution targets when light of wavelengths 450 nm and 650 nm was used. Bandpass filters for 450 nm (FBH450-10, Thorlabs) and 650 nm (FBH650-10, Thorlabs) were used to illuminate the test target with monochromatic light of wavelength 450 nm and 650 nm, respectively, by spectrally filtering the white light source of the optical microscope. The FZP lens was placed 7.2 mm away from the resolution test chart, and the real image of the chart magnified by a factor of 6 was observed with an optical microscope. In the case of 650 nm, an FZP lens was placed at 6 mm from the resolution test chart, and the real image of the chart magnified by a factor of 2.4 was observed under an optical microscope. The results of imaging groups 6 and 7 of the resolution test chart using 450 nm and 650 nm light are shown in Fig. S9a, c. For comparison, the results using the design wavelength of 550 nm light (the same graph as Fig. 5a in the main text) are shown in Fig. S9b. Although the resolution was slightly lower than for the design wavelength, each element could be confirmed. The results of the observation at 450 nm and 650 nm are shown in Fig. S9d-f. From the above, it was found that the FZP lens fabricated by this method can be used as a lens for imaging at wavelengths different from the design wavelength, although the resolution is slightly reduced when the lens is used at a wavelength different from the design

wavelength.

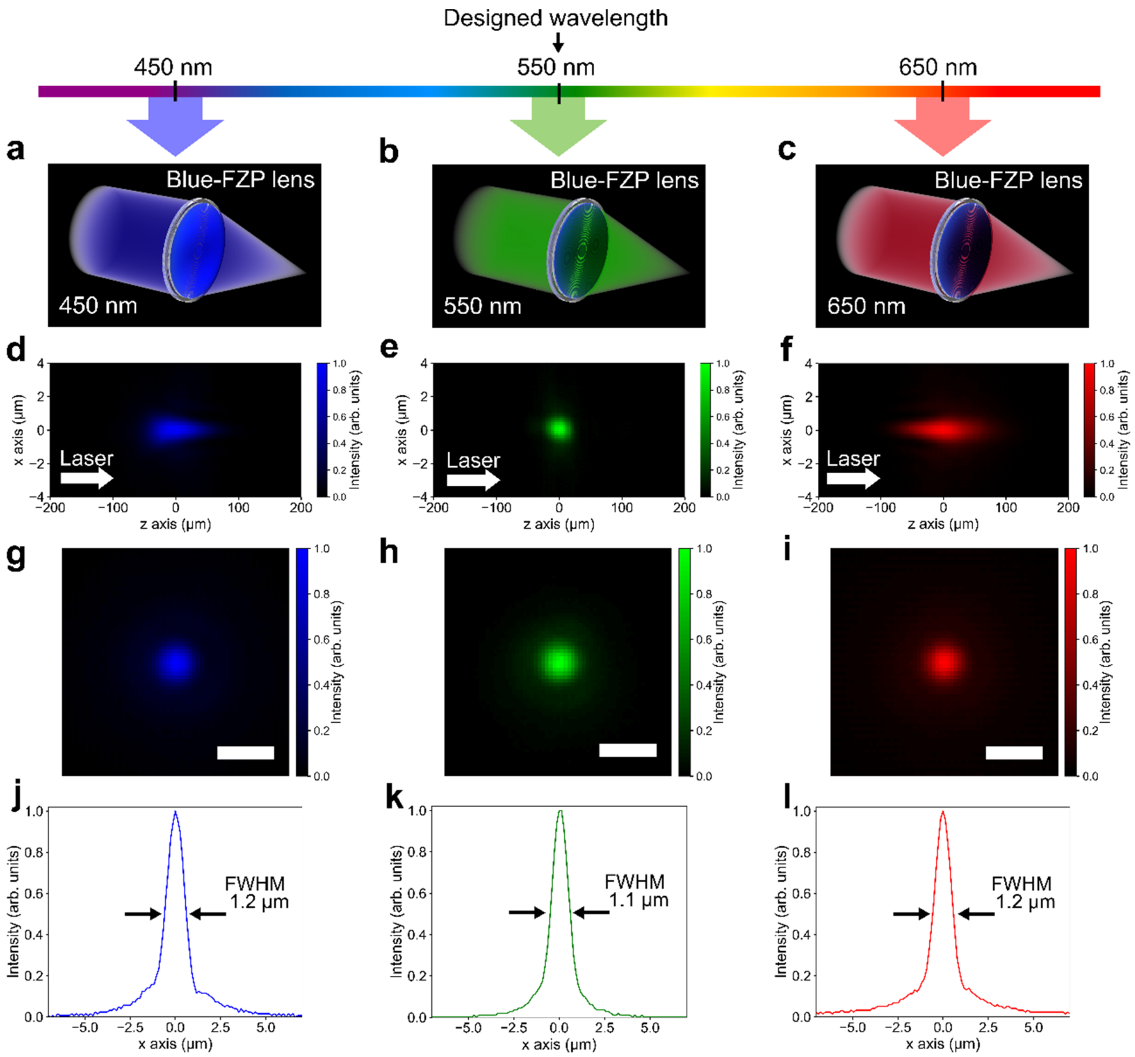

Fig. S8 Focusing profile measurements of laser beams of different wavelengths incident on an FZP lens with a design wavelength of 550 nm fabricated with blue resist. **a-c** Relationship between incident wavelength and the color resist of the FZP lens. **d-f** Results of focusing profile measurements in the xz plane. **g-i** Intensity profile at z=0. Scale bar size is 2 μm. **j-l** Cross-section intensity profile of **g-i**.

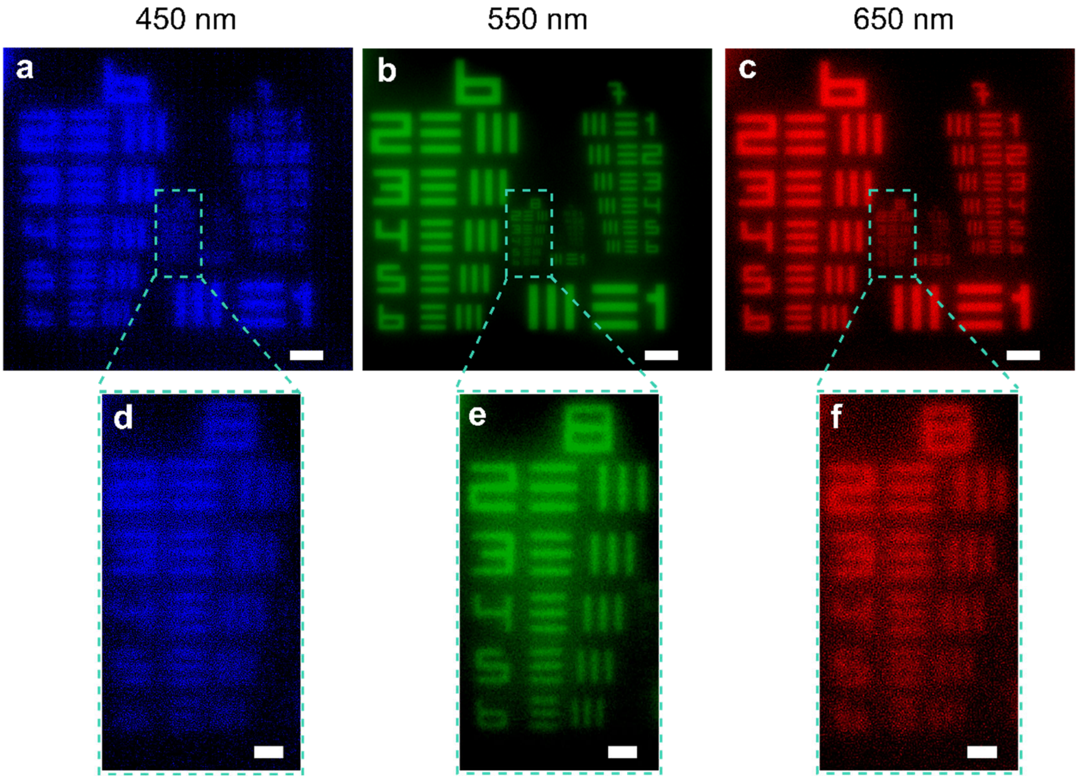

Fig. S9 Imaging results of USAF 1951 resolution targets using FZP lenses with a design wavelength of 550 nm. **a-c** Imaging results of Group 6 and 7 with incident light of 450 nm, 550 nm, and 650 nm, respectively. The scale bar size is 30 µm. **d-f** Imaging results of Group 8 with the area surrounded by the light blue dashed line in **a-c**. The size of the scale bar is 5 µm.

Supplementary Note 8: Evaluation of the thickness of FZPs.

This section describes the height of color resists. Fig. S10 shows the laser microscope images of the FZPs fabricated with blue, green, and red color resists. The evaluated thickness of the FZPs fabricated with blue, green, and red color resists are 0.49 µm, 0.51 µm, 0.47 µm, respectively.

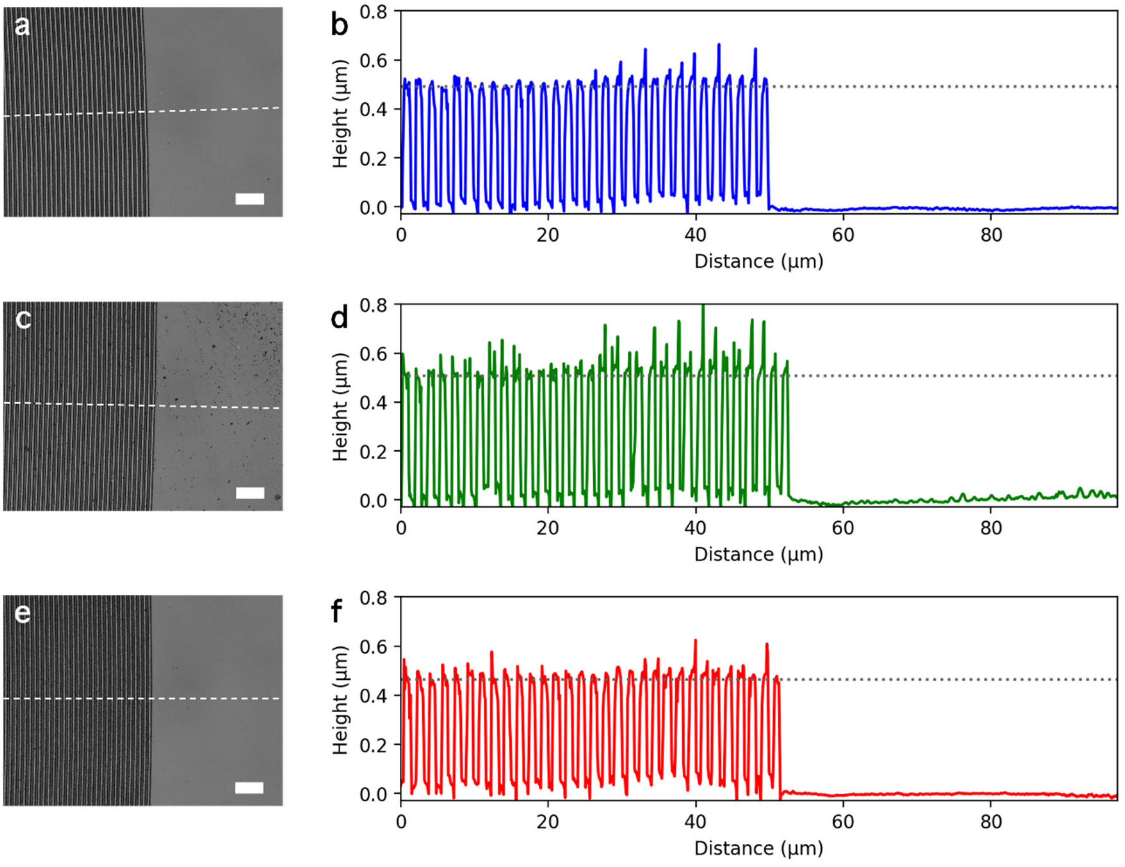

Fig. S10 FZP samples profiles measured using laser confocal microscope. **a, c, e** laser confocal microscope images of the FZP samples made with blue resist, green resist, and red resist, respectively. The size of the scale bar is 10 µm. **b, d, f** the structure profile measured along the dashed line on **a**, **c**, **e**. **b** The dotted line indicates the height of 0.490 µm. **d** The dotted line indicates the height of 0.508 µm. **f** The dotted line indicates the height of 0.465 µm.

Supplementary Note 9: Evaluation of current resolution limit of color resist structures

In this section, the resolution limits of color resist structures fabricated with our methodology are described. We fabricated different FZPs using a green resist (JSSG-9135) with the same fabrication method as outlined in the main text, where the designed structure diameters, and correspondingly, the minimal line width at the outer edge, were altered by modifying the mask pattern used during exposure. The design diameters were selected so that the apertures in the mask defining the outer line widths would have widths of 1.5 μm, 1.0 μm, 0.8 μm, and 0.6 μm. By observing how well the patterns were fabricated, the resolution limit of the current fabrication method could be gauged.

Laser microscope images of the correspondingly fabricated FZPs by the altered masks are shown in Fig. S11. For apertures from 1.5 μm to 0.8 μm, despite an expansion of approximately 0.25 μm in the lateral direction of the line patterns, the exposed and unexposed areas of the mask design was reproduced well by the presence and absence of the structures. The observed expansion of the resist pattern width is likely due to the effect of diffraction at the boundary between the aperture and mask causing exposure in parts of the masked regions [9], as well as the diffusion of curing initiators from exposed regions causing curing reactions to proceed in a finite area around the exposed areas [10]. At the smallest line width of 0.6 μm, resist remnants were observed to start to fill the unexposed areas due to this expansion. The expansion characteristics of the resist may potentially be corrected for by adjusting exposure intensity and mask size.

In our work, FZP designs with a minimum line width design of 0.8 μm width were selected for study as being representative of the finest spatial resolution achievable by this current method.

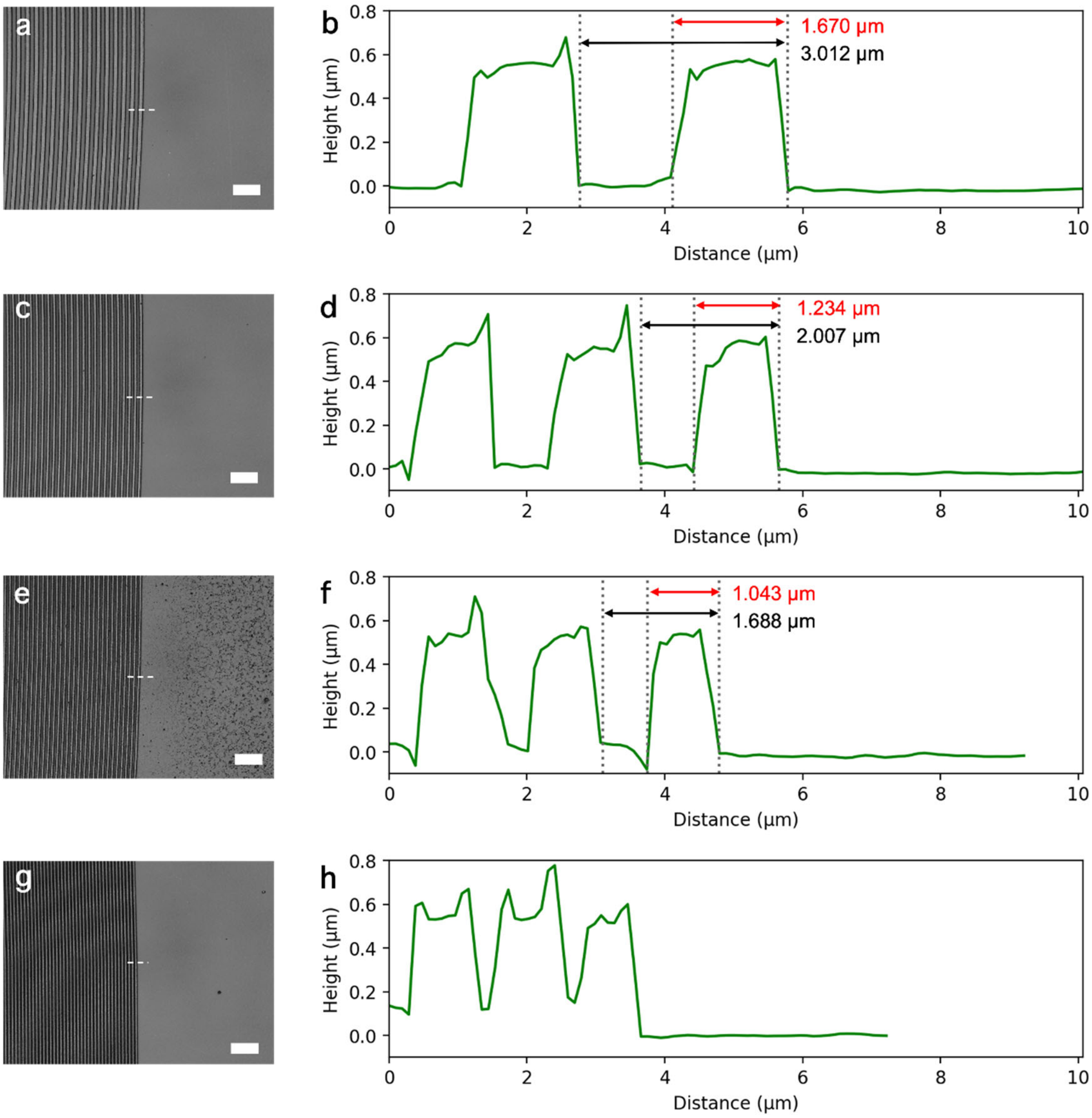

Fig. S11 Imaging results of resolution evaluation samples using laser confocal microscope. **a, c, e, g** laser microscope images of samples fabricated using the mask with aperture width of 1.5 µm, 1.0 µm, 0.8 µm and 0.6 µm, respectively. The size of the scale bar is 10 µm. **b, d, f, h** the structure profile measured along the dashed line on **a**, **c**, **e**, **g**. In **b**, **d**, **f**, Red arrows indicate the line width of the outermost structure, and black arrows indicate the width from the outermost structure to the second structure from the outside.

References

1. Li, Q.-K. et al. Multilevel phase-type diffractive lens embedded in sapphire. *Opt. Lett.* **42**, 3832-3835 (2017).
2. Li, X., Liu, F. Wan, R., Li, W. & Wang, P. Femtosecond laser-induced refractive index change and phase-type Fresnel zone plate in fluorotellurite glass. *Opt. Laser Technol.* **161**, 109216 (2023).
3. Wang, Z. et al. High efficiency and scalable fabrication of Fresnel zone plates using holographic femtosecond pulses. *Nanophotonics* **11**, 3081-3091 (2022).
4. Minerbi, E., Keren-Zur, S. & Ellenbogen, T. Nonlinear Metasurface Fresnel Zone Plates for Terahertz Generation and Manipulation. *Nano Lett.* **19**, 6072-6077 (2019).
5. Yoon, G., Jang, J., Mun, J., Nam, K. T. & Rho, J. Metasurface zone plate for light manipulation in vectorial regime. *Commun. Phys.* **2**, 156 (2019).
6. Attwood, D. *Soft X-Rays and Extreme Ultraviolet Radiation: Principles and Applications* (Cambridge University Press, Cambridge, 1999).
7. Far field projections in FDTD overview. Lumerical Support. https://optics.ansys.com/hc/en-us/articles/360034914713-Far-field-projections (2024).
8. Richards, C. A. et al. Hybrid achromatic microlenses with high numerical apertures and focusing efficiencies across the visible. *Nat. Commun.* **14**, 3119 (2023).
9. Smallwood, D. C., McCloskey, P., O'Mathuna, C., Casey, D. P. & Rohan, J. F. *Microsystems & Nanoengineering* **7**, 39 (2021).
10. Barouch, E., Hollerbach, U., Orszag, S. A., Allen, M. T., and Calabrese, G. S. Simulation of an advanced negative i-line photoresist, *Optical/Laser Microlithography* IV, 1463 (1991).
